# Contribution of community health workers to surveillance of vaccine-preventable diseases in the Obala health district

**DOI:** 10.11604/pamj.2017.28.207.11537

**Published:** 2017-11-07

**Authors:** Marius Zambou Vouking, Thierry Binde, Carine Nouboudem Tadenfok, Jean Marie Edengue Ekani, Daniel Ekra

**Affiliations:** 1 Center for the Development Best Practices in Health, Yaoundé Central Hospital, Henri-Dunant Avenue, Yaoundé, Cameroon; 2 Regional Unit of EPI Centre, Regional Delegation of Public Health, Yaoundé, Cameroun; 3 University of Buea, Department of Microbiology, Buea, Cameroon; 4 Ministry of Public Health, Yaoundé, Cameroun; 5 Université Félix Houphouët Boigny, Cocody, Abidjan, Cote d’Ivoire; 6 Direction of Expanded Program on Immunization, Abidjan, Cote d’Ivoire

**Keywords:** Contribution, community health workers, vaccine preventable diseases

## Abstract

**Introduction:**

The establishment of effective community-based surveillance is an essential objective of all disease surveillance systems. Several studies and reports have found that the situation is far from optimal in several developing countries such as Cameroon.

**Methods:**

We conducted a cross-sectional descriptive study to assess the contribution of community health workers to surveillance of vaccine-preventable diseases in Obala health district. The performance of community health workers was measured using: the number of cases referred to the health center, the percentage of accomplished referrals, the percentage of cases referred by community health workers confirmed by the staff of health centers. A questionnaire containing forty-seven questions (open-ended and closed-ended) was used for interviews with community health workers. The data were analyzed using SPSS 21 and Excel 2007. Counts and percentages are reported.

**Results:**

The study showed that the age ranged of community health workers was from 24 to 61 years with an average of 37.9 years ± 6.7 years. The most represented age group was between 40 and 50 with a percentage of 38.6%. The male sex was more represented than the female sex (61.4% vs 38.6%) or a sex ratio male man of 1.7. Forty-five percent of community health workers were selected at a village meeting, 93.1% of community health workers were involved in the surveillance of vaccine-preventable diseases and 87% experienced at least one preventable disease. Only 45.8% of them had the case definitions of the four diseases. Analysis of community health workers attendance at organized health committee meetings showed that 79% of community health workers attended at least one health committee meeting in 2015 and only 49% were monitored in 2015. Community health workers reported 42 suspected cases of measles, 37 of which actually went to the nearest Health Center, a baseline rate of 88%.

**Conclusion:**

Community health workers play a key role in the control of vaccine-preventable diseases in the Obala health district. Community-based surveillance is the foundation of surveillance activities. It is a mechanism based on simple case definitions of priority diseases and unexpected events or unusual conditions. Our study also reaffirms the importance of mastering case definitions and home visits and early detection of vaccine-preventable diseases.

## Introduction

Vaccination is one of the most cost-effective public health interventions to significantly reduce childhood mortality and morbidity [1]. Public health surveillance is typically defined as the ongoing, systematic collection, analysis, interpretation and dissemination of data regarding a health-related event for use in public health action to reduce morbidity and mortality and to improve health [2]. This is the principal instrument used in public health to recognize and manage spread of diseases and the data collected should be used in a timely manner for action, planning and evaluation and research to improve the health of the population [2]. The African region has had to deal with outbreaks of cholera, meningococcal meningitis, typhoid and influenza among other diseases in the last decade and lately the Ebola virus disease [3]. It is known that countries with weak surveillance systems or without community-based surveillance systems are not able to promptly detect and respond in a timely fashion to public health threats or events [3, 4]. There is a need then to strengthen disease surveillance at all levels and especially at the community level [3,4]. This study covered the Obala health district which recorded 819 cases of cholera in 2011 and also faced several epidemics of measles including some registered 160 cases in 2015 [5,6]. According to the World Health Organization (WHO), service delivery is the primary function of any health system and entails the provision of effective, safe, good quality care to those that need it with minimal waste and to address health care needs through promotion, prevention, treatment and rehabilitation [7]. Community Health Workers (CHWs) are men or women chosen by the local authorities and trained to provide health services at the community level [7]. CHWs are likely to be more sensitive to their fellow community members' health problems and to provide support to patients and their families [8,9]. This study aimed at establishing the contribution of CHWs of the Obala health district in the surveillance of vaccine-preventable diseases by analyzing their activities and determining their performance. More specifically, we have evaluated the knowledge, attitudes and practices of CHWs and analyze the performance on vaccine-preventable diseases.

## Methods

**Study setting** The study was conducted in the Obala health District, Centre region, Cameroon. The Obala health district covers over 675 km^2^ with 116,360 inhabitants distributed in 84 villages [6]. It covers two administrative units, including the Obala and Batchenga sub-divisions [6]. It is bounded on the North by the Mbandjock health district, on the west by the Esse health district, on the South by the Elig Mfomo health district, on the East by the Ntui and Sa'a health districts [6]. The Obala health District has 12 health areas, 32 health facilities including 3 hospitals, a Sub-divisional Medical Center, Catholic Medical Center and 27 Health Centers, 2 of which are non-functional [6]. The district has 90 health personnel, including 3 specialist physicians and 6 general practitioners. The urban center is home to a diverse population composed mainly of Beti (90%), Bamileké (7%) and a non-negligible Hausa community [6]. The Obala health district comprises 110 CHWs [6]. The dialogue structures are represented here by the health and management committees whose members are elected by the community [6].

**Study design**

From August to September 2016, we conducted a cross-sectional descriptive study on the CHWs in Obala health district. A forty-seven-item questionnaire containing open-ended and closed-ended questions was used to collect data from CHWs. The study questionnaire was verbally administered to the CHWs by four interviewers. The questionnaires were pre-tested in the near-by Djoungolo health area on 8 CHWs. We collected socio-demographic data (age and gender); work related data (number of houses visited per week, number of cases detected, number of cases referred); mode of appointment as CHW and other community health activities carried out. The CHWs performance in the control of vaccine-preventable diseases was measured in terms of the number of cases referred to the health center and the percentage of referrals accomplished. A confirmed case was defined as a probable case with evidence of acute flaccid paralysis, yellow fever, measles and neonatal tetanus.

**Participants:** All CHWs in the Obala health district were eligible to participate and were included if they consented. We interviewed 101 of the 110 CHWs in the health district.

**Statistical methods:** The data were analyzed using SPSS 21 and Excel 2007. The independent variables of age, gender, level in the health district system, role played in the chain of transmission of information on vaccine-preventable diseases were retained. The Dependent variables are close to relevant information on vaccine-preventable diseases, knowledge of vaccine-preventable diseases, mastery of case definitions and knowledge about the existence of community-based detection. Frequency measurements and averages of the variables were calculated with a confidence interval of 95%. The association measurements were made for the qualitative variables from the contingency tables where the X^2^ tests were carried out and the significance thresholds set at 5%.

**Ethics:** The study was approved by the ethics committee (number 00395 of 04 August 2016) of the Centre region and gate-keeper authorization was obtained from the Centre Regional Delegate of the Ministry of Public Health. Written informed consent was obtained from all participants before any interview was conducted.

## Results

### Characteristics of CHWs

There were 101 CHWs. Their age ranged from 24 to 61 years with an average of 37.9 years ± 6.7 years. Aggregated in age groups, the most represented class was that between 40 and 50 years with a percentage of 38.6%. The male sex was more represented than the female sex that is 61.4% against 38.6% or a sex ratio male man of 1.7. As concerns the level of education, 67.7% had attained the highest level of secondary education, 30.3% had primary education and 2% had no education. In terms of family status, 66.3% of respondents were married, 32.7% were single and 1% was widowed. In terms of monthly income, 59.4% of respondents had a monthly income of less than CFAF 36,270 compared to 38.6% with a monthly income of between 36,270 and 100,000. All of these characteristics are presented in Table 1.

**Table 1:** Socio-demographic characteristics of community health workers

Socio-demographic characteristics	Number	Percentage%
**Age class**		
[20;30[	9	8,9
[30;40[	37	36,6
[40;50[	39	38,6
[50;60[	14	13,9
60+	2	2
**Sex**		
Male	62	61,4
Female	39	38,6
**Health area**		
Batchenga	14	13,9
Efok	15	14,9
Ekabita-Mendoum	6	5,9
Endinding	4	4
Essong	11	10,9
Etoud-Ayos	8	7,9
Minkama	2	2
Nkolguem	9	8,9
Nkol-Mekok	5	5
Nkometou	9	8,9
Obala	11	10,9
Yemessoa	7	6,9
**Level of education**		
Not in school	2	2
Primary	30	30,3
Secondary	67	67,7
**Family status**		
Married	67	66,3
Single	33	32,7
Widower	1	1
**Monthly income**		
<36270	60	59,4
36270- 100 000	39	38,6
100 000 - 150 000	1	1
150 000 - 200 000	1	1

### Type of designation of community liaison officers

**Figure 1:**
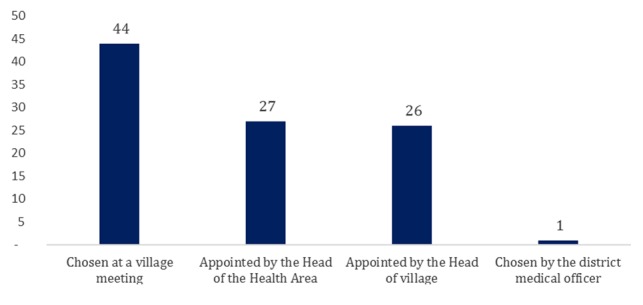
Distribution of community health workers according to their types of designation

CHWs were selected at a village meeting (45%), nominated by the head of the health area (28%), appointed by the village chief (27%) and chosen by the head of the village Neighborhood (1%). It emerged from this study that the method of election at a village meeting was the most represented at a rate of 45% (**Figure 1**).

### Knowledge of community health workers on vaccine-preventable diseases

**Figure 2:**
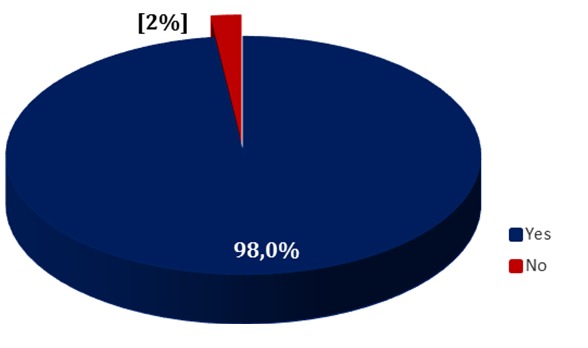
Knowledge of community health workers on vaccine-preventable diseases

Regarding the knowledge of CHWs on vaccine-preventable diseases, ninety-eight percent (98%) of the CHWs responding had a good knowledge of vaccine-preventable diseases (Figure 2). Women have a better knowledge of vaccine-preventable diseases than men, 100% versus 96.8%. Nkol Mekok and Obala health areas have the lowest knowledge levels of 80% and 90.9%, respectively. There was no significant difference between the age groups (p = 0.552), education levels (p = 0.820), monthly income (p = 0.987), gender (p = 0.987) (P = 0.297) compared to knowledge of Expanded Program on Immunization target diseases (Table 2).

**Table 2:** Breakdown of community health workers based on their knowledge of vaccine-preventable diseases

	Knowledge of EPI target diseases				p
	Yes		No		
	Number	%	Number	%	
**Age class**					
[20;30[	9	100	0	0,0	0,552
[30;40[	36	97,3	1	2,7	
[40;50[	39	100	0	0,0	
[50;60[	13	92,9	1	7,1	
60+	2	100	0	0,0	
**Sex**				
Male	60	96,8	2	3,2	0,257
Female	39	100	0	0,0	
**Health area**				
Batchenga	14	100	0	0,0	0,297
Efok	15	100	0	0,0	
Ekabita-Mendoum	6	100	0	0,0	
Endinding	4	100	0	0,0	
Essong	11	100	0	0,0	
Etoud-Ayos	8	100	0	0,0	
Minkama	2	100	0	0,0	
Nkolguem	9	100	0	0,0	
Nkol-Mekok	4	80	1	20,0	
Nkometou	9	100	0	0,0	
Obala	10	90,9	1	9,1	
Yemessoa	7	100	0	0,0	
**Level of instruction**				
Not in school	2	100	0	0,0	0,820
Primary	29	96,7	1	3,3	
Secondary	66	98,5	1	1,5	
**Family status**				
Marié	67	100	0	0,0	0,122
Married	31	93,9	2	6,1	
Single	1	100	0	0,0	
Widower				
< 36270	59	98,3	1	1,7	0,987
36270- 100 000	38	97,4	1	2,6	
100 000 - 150 000	1	100	0	0,0	
150 000 - 200 000	1	100	0	0,0	

**Figure 3:**
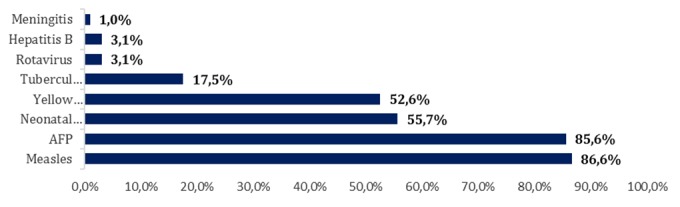
Distribution of knowledge of community health workers on vaccine-preventable diseases under surveillance in 2015

**Level of knowledge of community health workers on vaccine-preventable diseases:** Knowledge of CHWs on the various diseases preventable by vaccination under surveillance shows that measles is the best known disease at 87% followed by poliomyelitis at 86% of 56% neonatal tetanus and yellow fever at 53 % (Figure 3).

**Figure 4:**
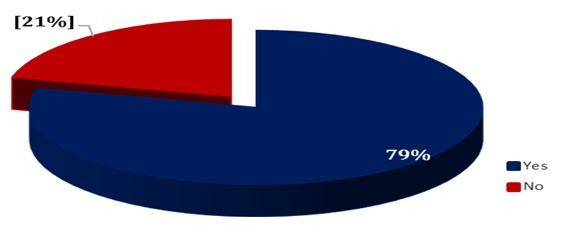
Participation of community health workers in organized health committee meetings in 2015

**Participation of community health workers in organized meetings of the health committee:** Analysis of the participation of CHWs in organized health committee meetings showed that 79% of CHWs attended at least one health committee meeting (Figure 4).

**Figure 5:**
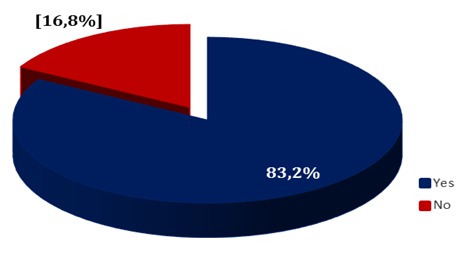
Intervention of community health workers in other health programs

**Intervention of community health workers in other health programs:** Eighty-three per cent (83%) of the community health workers reported responding to other health programs (Figure 5).

**Figure 6:**
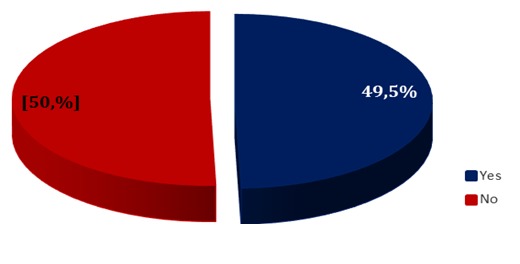
Supervision of community health workers by district management team and/or health area managers

**Supervision of community health workers:** Supervisions of health areas in the health district are done on a quarterly basis due to budgetary constraints. Forty-nine per cent (49%) of community health workers reported receiving at least one supervision from the head of the health area or from a senior staff member of the district management team in the year 2015 (Figure 6).

**Performance of community health workers:** Community health workers reported 42 suspected cases of measles, of which 37 actually went to the nearest health center, a baseline rate of 88%. Community health workers from all health areas reported at least one suspected case of measles. No suspected cases of neonatal tetanus, yellow fever and acute flaccid paralysis were detected in 2015 (Table 3).

**Table 3:** Number of suspected cases of measles detected by health areas in 2015

Health area	Suspected cases of measles in 2015	
	Detected	ReferredN (%)
Batchenga	4	3 (75)
Efok	5	4 (80)
Ekabita-Mendoum	3	3 (100)
Endinding	4	4 (100)
Essong	2	2 (100)
Etoud-Ayos	3	3 (100)
Minkama	2	2 (100)
Nkolguem	4	4 (100)
Nkol-Mekok	5	4 (80)
Nkometou	1	1 (100)
Obala	6	6 (100)
Yemessoa	3	2 (67)
District	42	37 (88)

## Discussion

This study indicates that community health workers are selected at village meetings (44.5%), appointed by heads of health areas (27.8%), appointed by village chiefs (26.7%) and chosen by the quarter heads (1%). It emerged from the study that designation at village meetings was the method of selection most used with a rate of 44.5%. This figure reveals that quite an important number of CHWs are not selected using the appropriate method because according to information obtained from the world health report on primary health care [10] and based on the various reports produced on the implementation of onchocerciasis control, CHWs must be appointed at a meeting of the health committee [11].

As concerns the involvement of CHWs in the fight against vaccine-preventable diseases, 93.1% of respondents said they were involved. This strong involvement can be explained by 1) the decentralization of surveillance in health districts since 2011 creating a situation whereby active surveillance is carried out at all levels of the health pyramid; 2) the poliomyelitis epidemic in Cameroon declared in October 2013 which led to the organization of 20 polio immunization campaigns between 2014 and 2016 incorporating an intensification of the call for detection of suspected cases of vaccine-preventable diseases in the communities [12]. Emphasis was placed on training community actors on case definitions of the vaccine-preventable diseases. It is important to note that there is a greater involvement of women to the rate of 94.9% compared to 91.9% for men. This sustain evidence that women are much more involved in health activities than men [9,13,14].

Eighty-three percent (83%) of the CHWs respondents reported their involvement in other health programs. Indeed, CHWs reported that apart from immunization campaigns they equally benefit from the distribution of Mectizan to discuss vaccine-preventable diseases with their populations. They also made simple field trips to find cases. Based on this consensual stakeholder diagnosis made in 2010, the Ministry of Public Health has decided to reform the deployment of community health workers with a view of harmonization [15]. In that regard, CHWs should be versatile in relaying health interventions beyond health facilities in a context marked by changes in the decentralization process that transfers responsibility for public health and related resources to local and regional authorities and the implementation of the National Health Development Plan.

Forty-nine percent (49%) of CHWs reported that they had received at least one supervision from the district health officer or district manager in the year 2015. This proportion is still not enough for a health system that intends to strengthen its impact on communities at the base. The inadequacy of supervisions was found that assessed the impact of training/supervision of CHWs in developing countries [9,16]. More so, the global initiative for the eradication of poliomyelitis has also shown that poliovirus is unlikely to continue to circulate in fully mobilized communities, even in the most difficult contexts [17,18].

A total of 42 suspected cases of measles were notified by CHWs, 37 of which actually went to the nearest health center, a reference rate of 88%. Furthermore, CHWs from all health areas have reported at least one suspected case of measles. The determinants of the performance of the CHWs understood as the combination of their accessibility, productivity, competence and reactivity are multiple [9,13]. Systemic factors take into account national policy directives, legal environment, allocated resources, planning and deployment of CHWs, communication, decision-making and control mechanisms [9,13,17]. Local factors are related to the availability of equipment and facilities, work teams, management of activities and the quality of interaction with health facility managers, peers and the communities. At the individual level, gender, marital status, personal ambitions, living conditions (culture, stability or social instability, conflict) and professional status determine the motivation to serve as CHWs [9,11]. The performance of a large-scale CHWs program requires goal-based planning, secure funding, government leadership, community support and effective mediation for concerted action [4,9,11]. This only goes to say that a strong government involvement is required [10].

## Conclusion

Community health workers play a key role in the control of vaccine-preventable diseases in the health district of Obala. Community-based surveillance is the foundation of surveillance activities. It is a mechanism based on simple case definitions of priority diseases and unexpected events or unusual conditions. As a matter of fact, our study reaffirms the importance of mastering case definitions and home visits and early detection of vaccine-preventable diseases.

### What is known about this topic

- Community health workers are likely to be more sensitive to their fellow community members' health problems and to provide to support patients and their families;
- Community health workers, by virtue of their proximity with the communities are able to play an important role in the detection and referral of cases of disease.

### What this study adds

- Community-based surveillance is the foundation of surveillance activities;
- Community health workers play a key role in the control of vaccine-preventable diseases in the health district of Obala;
- Our study also reaffirms the importance of mastering case definitions and home visits and early detection of vaccine-preventable dispenses.

## Competing interests

The authors declare no competing interests.
